# Supplementary material for: PFKFB3 exacerbates myocardial injury by accelerating CXCR4hi neutrophil mobilization after acute myocardial infarction
Source: PLoS One. 2026 Jan 29;21(1):e0333657. doi: 10.1371/journal.pone.0333657 (PMC12854469; doi:10.1371/journal.pone.0333657)
Supplement: S1 File — (DOCX) [file pone.0333657.s001.docx]

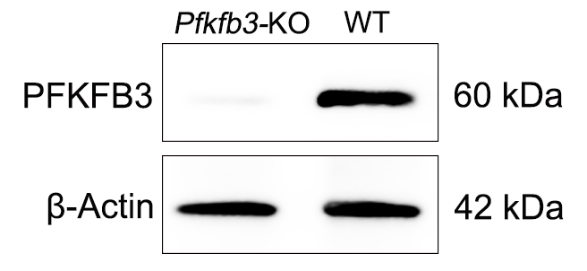


**Fig. S1** Western blot analysis of the levels of PFKFB3 protein in neutrophils isolated from WT and homozygous Neu-PFKFB3^-/-^ mice.


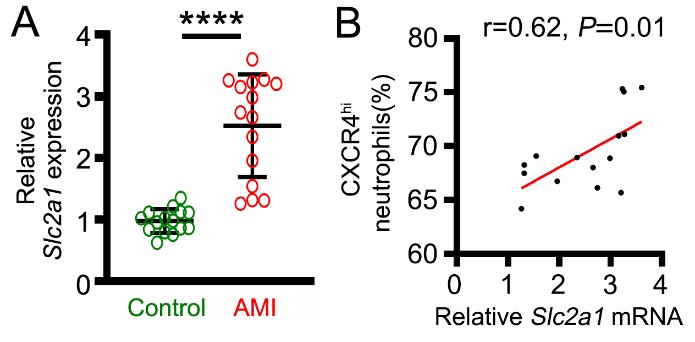


**S2 Fig.** Expression of *Slc2a1* gene. **A.** Expression of *Slc2a1* mRNA in the neutrophils of AMI patients. **B.** Correlation between *Slc2a1* mRNA expression in neutrophils and the percentage of circulating CXCR4^hi^ neutrophils in AMI patients.


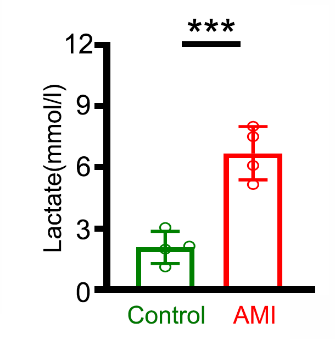


**S3 Fig.** Lactate production in AMI-neutrophils was significantly higher than those from healthy volunteers.
