# Supplementary material for: PFKFB3 exacerbates myocardial injury by accelerating CXCR4hi neutrophil mobilization after acute myocardial infarction
Source: PLoS One. 2026 Jan 29;21(1):e0333657. doi: 10.1371/journal.pone.0333657 (PMC12854469; doi:10.1371/journal.pone.0333657)
Supplement: Attachment — Submitted filename: Response to Reviewers.docx [file pone.0333657.s004.docx]

Reviewer #1: This study addresses a topic of clear clinical and scientific significance, focusing on neutrophils—key cells in the post-AMI inflammatory response—and their metabolic regulator, PFKFB3. The research design is systematic, progressing from clinical samples to animal models and cell-based experiments, which stepwise verifies the mechanism by which PFKFB3 promotes the mobilization of CXCR4hi neutrophils through glycolytic metabolic reprogramming. The conclusions are innovative and hold potential therapeutic relevance. However, certain aspects of the experimental details, data interpretation, and language expression require further refinement:

**Response:** On behalf of my co-authors, we thank you very much for your consideration of our manuscript entitled “**PFKFB3 exacerbates myocardial injury by accelerating CXCR4^hi^ neutrophil mobilization after acute myocardial infarction**” (**Manuscript ID:** **PONE-D-25-50535**). We greatly appreciate your comments. We have revised the manuscript accordingly, indicating the changes in the manuscript text file with blue. These changes have significantly strengthened and improved the manuscript.

1. Although NEU-PFKFB3 conditional knockout mice were utilized in this study, essential validation data—such as qPCR, Western blot, or immunofluorescence—are lacking and should be supplemented.

**Response:** Thanks for raising this important point.

In this study, neutrophils were isolated from homozygous Neu-PFKFB3^-/-^ mice. Then, the knockout efficiency for PFKFB3 was detected by western blot analysis. Results indicated that PFKFB3 protein was not expressed in the neutrophils of the homozygous Neu-PFKFB3^-/-^ mice (S1 Fig). We have updated “**AMI model construction**” in the “**Materials and methods**” section as below:

“The neutrophils were isolated from mice and detected by Western blot. We found that the neutrophils derived from Neu-PFKFB3^-/-^ mice did not express the PFKFB3 protein (S1 Fig).”

**S1 Fig. Western blot analysis of the levels of PFKFB3 protein in neutrophils isolated from WT and homozygous Neu-PFKFB3^-/-^ mice.**

2. In addition to HK-2 mRNA levels and ECAR measurements, it is recommended to include assessments of lactate production, ATP levels, or the expression/activity of key glycolytic proteins (e.g., GLUT1, PFK1).

**Response:** We appreciate your valuable suggestion.

This study is one of the serial studies that supported by the National Natural Science Foundation of China (82202389) and Postdoctoral Research Fund of Jinling Hospital (97103). Our team has already measured the expression levels of the key glycolytic proteins and metabolites. We found that the gene expression of *Slc2a1* (encoding glucose transporter 1, GLUT1) and the level of lactate (product of glycolysis) production were significantly increased in AMI patients compared with healthy volunteers (S2 and S3 Figs).

Following the reviewer’s suggestion, we have updated “**Glycolytic metabolism is required for PFKFB3-supported neutrophil CXCR4 expression**” in the **“Results”** section as below:

“Interesting, the gene expression of the facilitative glucose transporter member 1 (*Slc2a1*), which encodes the glucose transporter protein 1, also yielded similar results (**S2 Fig**).”

**S2 Fig. Expression of *Slc2a1* gene.** (A) Expression of *Slc2a1* mRNA in the neutrophils of AMI patients. (B) Correlation between *Slc2a1* mRNA expression in neutrophils and the percentage of circulating CXCR4^hi^ neutrophils in AMI patients.

**S3 Fig.** **Lactate production in AMI-neutrophils was significantly higher than those from healthy volunteers.**

3. Please more explicitly highlight the translational value of PFKFB3 as a potential therapeutic target in the Discussion section.

**Response:** We appreciate your valuable suggestion.

Following the reviewer’s suggestion, we have updated the discussion section in the revised version, thereby more clearly emphasizing the translational value of PFKFB3 as a potential therapeutic target. Details as follows:

PFKFB3, a famous glycolytic enzyme, is widely present in many immunocytes and plays a vital role in immunocyte inflammatory activation [20, 37-39]. Earlier investigations have shown that sepsis enhances glycolysis driven by PFKFB3 in macrophages, subsequently leading to proinflammatory polarization and inflammatory activation of these cells. Thus, specifically targeting the inhibition of PFKFB3-driven glycolysis in macrophages may serve as a promising therapeutic approach to prevent inflammatory damage in sepsis. Indeed, there are studies that corroborate this notion. In 2021, Xu and collaborators indicated that mice lacking myeloid Pfkfb3 are safeguarded from lung edema and cardiac dysfunction caused by LPS-induced endotoxemia [37]. Furthermore, in 2022, Yuan and his team demonstrated that apelin-13, an endogenous ligand for the angiotensin type 1 receptor-associated protein, mitigates LPS-induced inflammatory reactions and acute lung injury by suppressing PFKFB3-driven glycolysis in macrophages [40].

Consistent with macrophages, our previous studies revealed that PFKFB3 plays as an accelerator in the inflammatory activation of neutrophils during sepsis [18].
