# Supplementary material for: PFKFB3 exacerbates myocardial injury by accelerating CXCR4hi neutrophil mobilization after acute myocardial infarction
Source: PLoS One. 2026 Jan 29;21(1):e0333657. doi: 10.1371/journal.pone.0333657 (PMC12854469; doi:10.1371/journal.pone.0333657)
Supplement: Attachment — Submitted filename: Response_to_Reviewers_auresp_2.docx [file pone.0333657.s005.docx]

Reviewer #1: The author appears to have responded to all my questions. However, I am unable to view the supplementary figures (Figure S1-S3) mentioned in the response through the review system. Could you please send these images to me for review?

**Response:** On behalf of my co-authors, we thank you very much for your consideration of our manuscript entitled “**PFKFB3 exacerbates myocardial injury by accelerating CXCR4^hi^ neutrophil mobilization after acute myocardial infarction**” (**Manuscript ID:** **PONE-D-25-50535R1**). We greatly appreciate your comments. The supplementary figures (Figure S1-S3) had been presented as following. Once again, we would like to thank you for your careful review which has helped us improve the scientific rigor and reliability of the manuscript.

**Fig. S1** Western blot analysis of the levels of PFKFB3 protein in neutrophils isolated from WT and homozygous Neu-PFKFB3^-/-^ mice.

**S2 Fig.** Expression of *Slc2a1* gene. **A.** Expression of *Slc2a1* mRNA in the neutrophils of AMI patients. **B.** Correlation between *Slc2a1* mRNA expression in neutrophils and the percentage of circulating CXCR4^hi^ neutrophils in AMI patients.

**S3 Fig.** Lactate production in AMI-neutrophils was significantly higher than those from healthy volunteers.
